# The impacts of COVID-19 on older adults in Uganda and Ethiopia: Perspectives from non-governmental organization staff and volunteers

**DOI:** 10.1371/journal.pgph.0003691

**Published:** 2024-09-04

**Authors:** Satveer Dhillon, Isaac Luginaah, Susan J. Elliott, Justine Nagawa, Ronah Agaba Niwagaba

**Affiliations:** 1 Department of Geography and Environment, Western University, London, Ontario, Canada; 2 Department of Geography and Environmental Management, University of Waterloo, Waterloo, Ontario, Canada; 3 Reach One Touch One Ministries, Mukono, Uganda; African Population and Health Research Center, KENYA

## Abstract

The COVID-19 pandemic had a substantial impact on older adults, especially in Sub-Saharan Africa (SSA). To support older adults during this time, non-governmental organizations (NGOs) coordinated programs to help provide for basic needs related to food and water security and healthcare. This research explores the attitudes, perceptions and experiences of NGO staff and volunteers who provided support to older adults in SSA in rural East Africa during the COVID-19 pandemic. In-depth interviews (n = 28) were conducted with NGO staff and volunteers in Uganda and Ethiopia between September and December of 2022. Overall, NGO staff and volunteers reported high levels of knowledge surrounding the COVID-19 pandemic and stated that one positive of the COVID-19 pandemic was the improved hygiene practices. However, the NGO staff and volunteers also reported that the pandemic and the associated public health measures exacerbated pre-existing social inequalities, such as increasing pre-existing levels of food insecurity. The exacerbation of pre-existing social inequalities may be one reason for the increased reliance on NGO services. The learnings from the COVID-19 pandemic and associated public health measures can be utilized to create targeted strategies to mitigate the negative impacts of future public health crises on vulnerable populations.

## Introduction

During the COVID-19 pandemic, older adults, defined as individuals 60 years or older [1] were significantly impacted not only by the virus but also the public health measures implemented by various levels of governments worldwide. Those who were older had more likelihood of severe COVID-19 outcomes due to both age and the presence of underlying illnesses in some individuals [2–4]. Indeed, more than 80% of COVID-19 deaths worldwide occurred among those 60 years or older [5]. Along with the severe outcomes of the virus itself, public health measures (e.g., social distancing and lockdowns) had consequences for older adults (e.g., increased loneliness and social isolation) [6–8]. These impacts, while experienced on a global scale, were geographically and spatially varied. For example, from January 2020 to December 2021, there were an estimated 4.74 million excess deaths in India and 1.02 million excess deaths in Indonesia [9]. Also, Sub-Saharan Africa (SSA) experienced difficulties in COVID-19 vaccine administration due to the lack of equitable access and challenges in distribution [10]. Lockdowns in SSA negatively impacted livelihoods, further exacerbating food insecurity and other pre-existing social inequalities [11, 12]. These impacts were heightened among the older adult population [13–15]. For this reason, the United Nations identified older adults, particularly older women, as experiencing the highest degree of marginalization as a result of the pandemic [16].

Older adults in SSA are the fastest-growing aging population in the world [17, 18]. Despite this, there is little financial, social, healthcare or other forms of support for this vulnerable population [19, 20]. For example, older adults have limited access to pensions and/or healthcare [21]. Consequently, older adults are reliant on financial support from families and non-governmental organizations (NGOs), where and when available [22].

During the pandemic, NGOs worked to fill the void left by the lack of state programs in many parts of the world. For example, in Brazil, civil society organizations adjusted their service delivery from development to providing basic needs related to food and water security [23]. SSA has a history of similar responses to crises. For example, NGOs have been an essential part of the response to HIV/AIDS on the continent [24]. To respond to the needs of older adults during the COVID-19 pandemic, NGOs delivered services related to food and water security, pandemic preparedness as well as basic health services [25–27]. Several NGOs in SSA experienced increased demand, indicating the dependence communities have on these organizations [25]. Staff and volunteers at NGOs in low- and middle-income countries (LMICs) were central in coordinating services in response to the pandemic [28]. Due to their first-hand experience in providing support during crises, NGOs were in a good position to offer valuable insights into understanding how the pandemic had impacted vulnerable communities, such as older adults, as was the case in Uganda and Ethiopia.

Located in East Africa, Uganda and Ethiopia have populations of 48.6 million [29] and 123 million, respectively [30]. In Uganda, the number of older persons in 2020 was 1.5 million, and that number is expected to increase to 6 million by 2050 [31], which will represent 12.5% of the total population [32, 33]. By 2050, the older adult population in Ethiopia is also expected to increase from nearly 5% of the total population (around 4 million) to 10% (or around 19 million) [34, 35]. While higher-income countries already have substantial older adult populations with systems to support them, lower-income settings do not [21, 36, 37]. Further, due to the HIV/AIDS pandemic, these older adults are often raising the next generation of orphaned children, many of whom are also HIV positive [21, 38]. Compounded with a lack of pensions, universal access to primary education as well as healthcare, older adults often have to work and/or rely on NGOs to meet their daily needs [21, 38–40]. During the COVID-19 pandemic and similar to other countries in SSA and around the world, older adults residing in these countries experienced stringent lockdowns, such as curfews and closure of public transportation [41, 42]. These lockdowns impacted their ability to access basic needs, such as water, food and healthcare [15, 43, 44]. As a result, NGOs, such as Reach One Touch One Ministries (ROTOM), provided extensive support. ROTOM was founded in 2004 in Uganda (2014 in Ethiopia) in response to the challenges existing among the older adult population. Currently, ROTOM has four international partners (Canada, USA, Germany, UK) responsible for providing funding and support. ROTOM supports the health and social care needs of older adults and their dependents in Uganda and Ethiopia by providing them with healthcare, water and sanitation, food security and educational access for grandchildren [45].

This paper reports the results of a qualitative investigation of the attitudes, perceptions and experiences of ROTOM staff and volunteers who provided support to older adults in Uganda and Ethiopia during the pandemic. This work is informed by a Political Ecology of Health (PEH) framework, which aims to connect large-scale economic, political and social influences to health and well-being outcomes at a local level [46–49]. In this sense, our research examines how social and political factors, such as public health measures, impacted vulnerable communities in Uganda and Ethiopia during the pandemic. PEH also looks at the conditions that shape transmission patterns and disease vulnerability [47]. Our research helps to uncover social inequalities that were produced or exacerbated during this time and may have impacted vulnerability to the pandemic. Furthermore, this framework guides our examination of different local understandings of COVID-19, represented by a group of staff and volunteers who played various support roles during the pandemic.

## Research design and methods

### Ethics statement

The study was reviewed and approved by Western University’s Non-Medical Research Ethics Board (NMREB) (Project ID: 121196) and ROTOM. All study participants had given their verbal informed consent to participate in this study.

Qualitative in-depth interviews (n = 28) with staff and volunteers from ROTOM were conducted between September and December of 2022 to investigate attitudes, perceptions and experiences when providing support to older adults during the pandemic. All participants had worked on the ground during the COVID-19 pandemic and held various roles (Table 1). Village volunteers were responsible for providing support for ROTOM-related activities on the ground, such as visiting older adults. Field officers were staff members primarily responsible for coordinating ROTOM-related activities and providing updates to the leadership team. 26 individuals were from Uganda, while 2 were from Ethiopia. Interviews were conducted until saturation was reached, defined as when collecting additional data does not produce any new insights [50, 51]. Ethical clearance was provided by Western University (located in London, Ontario, Canada) and ROTOM. Additional information regarding the ethical, cultural, and scientific considerations specific to inclusivity in global research is included in the Supporting Information (S1 Checklist).

**Table 1:** Categories of interviewees in ROTOM.

Categories	Number (%)
Leadership (Board Member, Senior Staff, Executive Directors)	9 (32%)
Village Volunteers	6 (21%)
Healthcare (Doctors, Nurses)	4 (14%)
Special Projects (Agriculture initiatives, ROTOM school)	4 (14%)
Field Officer	3 (11%)
Evangelist	2 (7%)
**TOTAL**	28 (100%)

Potential participants received a letter of information explaining the research objectives and potential risks and benefits and inviting them for an interview. The response rate was 93%. Interviews were conducted in English virtually through Zoom between September 2022 to December 2022. Each interview began with an overview of the letter of information and consent process before commencing recording. An interview guide was used to examine the impact of COVID-19 on older adults from the perspectives of NGO staff and volunteers (S1 Text). Topics included their understanding of the COVID-19 pandemic, the impact of the pandemic and associated public health measures on older adults, and COVID-19 vaccine uptake. On average, interviews lasted 36 minutes and ranged from 19 minutes to 1 hour 5 minutes.

Interviews were transcribed verbatim and anonymized. The transcripts were then uploaded into NVivo for thematic analysis. A codebook was developed using deductive and inductive themes for line-by-line coding. The themes and codes, along with the transcripts, were reviewed multiple times to ensure consistency. Once results were drafted, they were sent to all participants to provide them with the opportunity to review the results, validate the data and create opportunities to add additional information [52]. 6 participants provided further feedback.

## Results

Four key themes emerged from the interview data: 1) COVID-19 knowledge and perceptions among NGO staff and volunteers, 2) the negative impacts of the COVID-19 virus, 3) the impacts of the associated public health measures, and 4) improved hygiene: the silver lining of the COVID-19 pandemic. Participants’ quotes are used to punctuate the findings.

### COVID-19 knowledge and perceptions among NGO staff

Most participants had a clear understanding of how the COVID-19 virus impacted individuals, including the signs and symptoms of COVID-19. Several NGO staff and volunteers stated that the COVID-19 virus is airborne:

“*And it [the COVID-19 virus] is very dangerous; it spreads through the air, through touch, and it’s very highly contagious and very dangerous*” (P_20,_ NGO Uganda Leadership).

At the time of the interview, 82% (n = 23) of participants believed Uganda or Ethiopia was not COVID-19-free as individuals continued to have signs and symptoms and were testing positive. Contrarily, 18% of the participants (n = 5) indicated that Uganda or Ethiopia were COVID-19-free at the time of the interview as they stopped hearing about new COVID-19 cases.

The majority of the NGO staff and volunteers learned about COVID-19 through the radio and other news sources, such as television, social media, and the newspaper. Some participants heard about COVID-19 before it entered Uganda:

“*On the news, we first heard it when it wasn’t in Uganda. We heard it on CNN on the television and then also on the radio here in Uganda. Then, through the Ugandan website, the Ministry of Health gave us updates on the COVID-19 virus. […] So, it was very scary. It’s very scary”* (P_27_, NGO Uganda Village Volunteer).

Participants also highlighted a number of key challenges for older adults arising due to the COVID-19 pandemic, including psychosocial effects and vaccine hesitancy.

NGO staff and volunteers mentioned how the COVID-19 pandemic, including the fear of the virus, increased older adults’ worry. Research showed that older adults were more likely to have severe outcomes, and as a result, “*they could not believe that they could survive”*.

It was noted that older adults were living with lingering fear as every time they felt unwell, they thought, “*Is it something that is going to take away my life*?*”* P_6,_ a village volunteer, noted that this was not the case before the COVID-19 pandemic. These feelings of fear may have been intensified due to lived experiences with the HIV/AIDs epidemic and recent crises such as Ebola.

During the COVID-19 pandemic, older adults also lost their loved ones, negatively impacting their mental wellbeing. Some of the loved ones who passed away due to the virus were the older adults’ financial and psychological support, and as mentioned by a Field Officer:

“*Now they [the older adults] think about those children when they are in need, and they cry and say, “Oh, I wish my child was around. He, or she could have supported me through this challenge””* (P_1,_ NGO Uganda).

Participants also noted that older adults were hesitant to receive the COVID-19 vaccine due to misinformation:

“*Those uncomfortable with the vaccine, hearing from others how they’re going to become either deaf or they will lose their sight or get strokes. So, some of them were really worried and anxious. Whenever they felt like they were not feeling well, they would attribute it to the vaccine”* (P_25_, NGO Uganda Special Projects).

“*They said they were going to die*, *so they will not come for vaccination*. *And we have some people here in Uganda who do not accept the use of the vaccine*. *They believe that the vaccine is somehow satanic*. *They will not allow people to take it*. *They discouraged people from going for vaccination*. *Other people believe that once you are vaccinated*, *you are going to die within a month*. *So it was difficult to convince the older people to come for the vaccine”* (P_1_, NGO Uganda Field Officer).

### Challenges and potential consequences of public health measures

All participants noted that the public health measures implemented during the pandemic, such as wearing a mask, social distancing, and lockdowns, had negative consequences on the lives of older adults. For example, NGO staff and volunteers stated that, at times, it was difficult to convince older adults to wear masks. Furthermore, older adults, especially those with other health conditions, had difficulties wearing a mask as “*they felt they were being suffocated*.*”* Further, older adults with dementia had compounding difficulties as they would “*be home and forget and remain with the mask*”.

Social distancing also aggravated loneliness among older adults as in-person gatherings were prohibited. As stated by a participant on the leadership team:

“*Africans and Ugandans, in particular, are very social people. Most of these people [referring to older adults] do not have something like a TV or they cannot read. They don’t have phones. So they were really pushed into a lonely corner when the isolation came”* (P_3,_ NGO Uganda).

It was also underscored that older adults might not be able to socially distance as they share a room with “*five or six people”*.

During the COVID-19 pandemic, several existing challenges were also exacerbated. Participants stated that older adults and their family members lost employment during this time. As a result, it was difficult for them to afford essentials, further aggravated by inflation and increased cost of living. Additionally, because of these economic challenges and several other factors, such as lockdowns, food insecurity became a major concern:

“*So I can say before, there were a lot of elders who couldn’t get two meals a day, but during COVID, when we checked with the elders, they only had one meal the whole day. So, we try to give something to our elders, but there are a lot of elders without any support, or any income. So they are struggling with food insecurity”* (P_24_, NGO Ethiopia Leadership).

Older adults were also afraid of visiting their gardens to access food due to law enforcement and public health measures. Another factor impacting food insecurity was reverse migration, which was a common occurrence during the pandemic as those who were working in cities moved back to their villages due to the lockdowns. This phenomenon increased the burden on older adults:

“*And then there is a food problem because the household numbers had doubled. And so there are many more mouths to feed, and the seniors that we support, or generally seniors in Uganda, are weak and cannot grow food on their own. And so even the little food they had grown to take them through the time was not enough. So, there was a very huge need for food. I think those two were the main issues that came out”* (P_26_, NGO Uganda Leadership).

In addition, the lockdowns and closure of public transportation had a determinantal impact on access to healthcare:

“*With regard to healthcare*, *it was also challenging a bit*. *Since most transport means were closed*, *they could not access medical facilities anymore*, *and there were mobility challenges*. *It was really hard for all the people who were depending on* [prescription] *refills*, *regular checkups*, *and regular visits to the health centers”* (P1, NGO Uganda Field Officer).

### Increased poverty for older adults

Over half of the respondents (54%) reported that the needs of older adults had changed as a result of the pandemic; older adults can no longer meet basic needs, which is compounded by the lack of governmental support:

“*Because in Ethiopia, there is no pension for all people. I mean, when you want to get a pension, you must have used to work formally in the government sector. […] Most of our elders, they used to be daily labourers or maid servants. Now, they do not have any pension, and most of them, when they were working as a daily labourer, they only used that money for daily needs, and they did not make enough to save that income. So, at this age, they do not have any savings or no pension or any income. They lean on either their children, relatives, well-wishers, non-governmental, or non-governmental organization support. So now the well-wishers, I mean most of the Ethiopian people, are struggling with the living costs”* (P_24_, NGO Ethiopia Leadership).

“*I think in Uganda*, *we still have a long way to prioritize older persons and their needs because I believe that they’re not really prioritized*. *[…] It was very evident during COVID-19 that actually the people that were most susceptible to the disease were older people*” (P_26_, NGO Uganda Leadership).

NGO staff and volunteers also illustrated that the organization cannot support all the needs of older adults, especially with increasing costs. Older adults require more support due to several contributing factors, including the older adults’ relatives losing their jobs and reverse migration. Even though the severity of the pandemic has decreased, *“Surviving itself is still so hard for them [older adults]”*.

### Improved hygiene: Silver lining of the COVID-19 pandemic

While the COVID-19 pandemic caused “*more harm than good*,*”* there were a few positives that emerged from this historical time period. Participants noted that there were better hygiene practices, resulting in improved health outcomes and increased confidence for other public health crises (e.g., Ebola) and illnesses (e.g., typhoid):

“*One, there was an increase in awareness of health. And, with that, I will start with the basics, like hand washing. Initially, people did not take hand washing as a serious thing, but then with COVID-19, we had to create awareness. We had to teach the older persons how to do proper hand washing, which also helped to reduce other infections, especially diarrhea. So that was a good thing”* (P_12_, NGO Uganda Healthcare).

Further, NGO staff and volunteers reported that as a result of them encouraging older adults to follow the public health measures, they were protected against the COVID-19 virus. Masks were described as a “*saving instrument*.” Other public health measures also had similar positive impacts:

“*Yeah, it’s social distancing, like staying at home, wearing masks, washing hands. Those prevention measures really helped because we didn’t lose many seniors because of COVID-19. We really encouraged them to stay at home by using our volunteers. They were encouraged to receive the vaccination. We helped them”* (P_2_, NGO Uganda Leadership).

## Discussion

This research explored the attitudes, perceptions and experiences of NGO staff and volunteers who provided support to older adults in SSA during the COVID-19 pandemic. We found, firstly, that the NGO staff and volunteers reported a good understanding of the signs and symptoms as well as the impact of the COVID-19 pandemic globally and modes of transmission. These findings are consistent with several studies conducted in SSA [53–56], focused on healthcare workers’ understanding of the COVID-19 pandemic. A better understanding of the COVID-19 pandemic can result in improved outcomes for the individuals supported by these NGOs [57, 58].

Secondly, by using the PEH framework, our findings underscored how public health measures negatively impacted health and wellbeing outcomes at a local level [47]. For instance, the research participants reported that the older adults they supported felt socially isolated, a finding that has been highlighted in other studies conducted in both LMICs, such as Ethiopia [44], Uganda [15], the Philippines [59] and high-income countries (HICs), such as Austria [60], the UK [61], and the USA [62]. Further, the PEH framework looks at the conditions that shape disease vulnerability [47]. For instance, in LMICs, such as Uganda and Ethiopia, older adults were unable to connect with others using technology, further isolating them [15]. However, in HICs, older adults were able to use technology, such as Zoom, to maintain connection to their friends and family [62]. The ban on public transportation in LMICs further limited access to healthcare for older adults, as stated by several NGO staff and volunteers. In HICs, however, initiatives such as telehealth were utilized to help connect healthcare service providers to individuals [63]. These differences highlight the need for NGOs in LMICs to implement initiatives where healthcare services are brought closer to vulnerable communities. One example would be to utilize field nurses to provide care in the communities.

Negative impacts on mental wellbeing due to the pandemic were frequently highlighted by NGO staff and volunteers. These findings align with several other studies illustrating that due to social isolation, fear of the virus and exposure to the media contributed to distress [14, 64, 65]. Scholars have also noted that within SSA in particular, a higher incidence of depression is reported among older adults when compared to younger adults [66]. Despite this, there is a paucity of mental health services for older adults within SSA [67–69]. Local interventions, such as training community health workers and NGO staff and volunteers to provide mental health education and counselling, can be implemented to help improve the mental well-being of this population [14, 70].

Misinformation surrounding the vaccine was a concern for NGO staff and volunteers. While this was a global concern [71, 72], within the context of SSA these concerns were likely amplified due to the history of unethical research practices conducted by pharmaceutical industries in Africa [73]. Further, hesitancy towards the vaccine may be attributed to the general distrust people residing in SSA and the global public have towards the government, along with any negative experience they might have had with the healthcare system [74]. Hence, there is a need, especially for future public health crises, to dismantle hesitancy by building trust within communities by involving key stakeholders, such as local healers, religious leaders, NGOs, and community members in vaccine campaigns [73].

The economic challenges in Uganda and Ethiopia among older adults and, for that matter, in the majority of SSA, have exacerbated pre-existing social inequalities. During the COVID-19 pandemic, the closure of markets, lockdown measures, and the global negative impacts on businesses have caused severe socioeconomic impacts [75]. Our study also found that as a result of reverse migration, there was an increasing number of individuals depending on older adults, who themselves were struggling to afford basic needs, such as food and water. Despite these challenges, it is clear from participants and the relevant research literature that there are little financial or other types of support available for older adults in SSA [19, 20, 76, 77]. Compounded with the diminishing of the traditional family system, older adults are often left to their own means [78]. Hence, it is imperative for the government to provide increased financial support, such as pensions [79–81] and to work alongside NGOs, such as ROTOM, to better support older adults.

Finally, our research showed the importance of continuous education surrounding sanitation practices, such as handwashing. By wearing masks and frequently washing hands with soap and water, there has been a decline in other (respiratory) infectious diseases [82, 83]. Good hygiene practices can protect against other infectious diseases, such as Ebola [84]. However, in SSA, only 33.5% of households with an observed handwashing place at home have water and soap [85]. These findings underscore the importance of continued education on better hygiene practices and the need to focus on access to safe water and sanitation.

## Conclusion

This research focused on the attitudes, perceptions and experiences of NGO staff and volunteers who provided support to older adults in SSA during the COVID-19 pandemic. Through the political ecology of health framework, we identified several findings related to the negative impact of the COVID-19 virus among older adults and the challenges that occurred due to the public health measures. The COVID-19 pandemic exacerbated pre-existing social inequalities and increased poverty. Consequently, the NGO is also seeing an increased demand for services.

A potential limitation of this research is the lack of older adults’ voices within this study. During the time of the interview, there were several barriers, such as the COVID-19 pandemic and Ebola cases, that made it difficult to interview older adults residing in Uganda and Ethiopia. With the decrease in COVID-19 cases, it is crucial to interview older adults to gain insights into their perceptions of the pandemic’s impact and assess the support provided by NGOs during this period. Another limitation is that only two individuals from Ethiopia were interviewed due to ROTOM Ethiopia’s team being smaller. Finally, another key next step would be to understand the strategies implemented by NGOs during the COVID-19 pandemic. By doing so, a set of strategies can be recommended and implemented by other NGOs during public health crises to support vulnerable populations, such as older adults.

Nonetheless, our research highlighted the experiences of NGO staff and volunteers when providing support to older adults during the pandemic. As discussed, there are several negative impacts of the pandemic and associated public health measures, thus illustrating the importance of using targeted approaches to decrease the negative impact of public health crises on older adults. Considering the rapidly aging population in SSA, increased attention needs to be paid to this vulnerable population in policy and practice, especially during future public health crises.

## Supporting information

S1 Checklist PLOS’ questionnaire on inclusivity in global research. (DOCX)

S1 Text Interview guide. (DOCX)
