# Supplementary material for: The impacts of COVID-19 on older adults in Uganda and Ethiopia: Perspectives from non-governmental organization staff and volunteers
Source: PLOS Glob Public Health. 2024 Sep 4;4(9):e0003691. doi: 10.1371/journal.pgph.0003691 (PMC11373809; doi:10.1371/journal.pgph.0003691)
Supplement: S1 Text — (DOCX) [file pgph.0003691.s002.docx]

**The impact of COVID-19 on Older Adults and Strategies to Rebuild after the COVID-19 Pandemic**

**Individual Interview Guide**

**General Questions**

1. What do you know about the COVID-19 virus? (Prompt: mode of transmission, treatment, etc.)
2. How did you come to know about the COVID-19 outbreak?
3. In your opinion, is Uganda or Ethiopia COVID-free? (Prompt: why/why not)

**Impact of the COVID-19 pandemic on older adults**

1. How would you describe the impact of the COVID-19 virus on older adults?
2. Could you describe your views about how public health measures related to COVID-19 impact older adults?
3. How is the vaccine uptake amongst older adults?
4. Could you talk about any social support services that were available for older adult COVID-19 victims and survivors?
5. How would you describe any positive and/or negative changes you may have experienced/observed after the COVID-19 pandemic? (Prompt: relationship of people to survivors and their families, banning of dangerous cultural practices, etc.)
6. How would you describe the impact of COVID-19 on the mental health of older adults?

**The role and strategies of non-profit groups in curtailing the COVID-19 pandemic**

1. How were you involved in the fight against the COVID-19 pandemic?
2. Please describe the strategies you and /or your organization adapted to curtail the virus?
3. Could you describe the lessons learned and how you may approach a different outbreak?
4. What support mechanism (e.g., COVID-19 supplies/equipment) did you receive from the Ugandan/Ethiopian government, and local and international non-governmental organizations to help you cater for COVID-19 victims during the pandemic?
5. Could you describe what government mechanisms are currently put in place that would help to prevent or minimize the impact of any potential pandemic?
6. What is your organization currently doing differently compared to before the pandemic?
7. What is your organization currently doing to prepare themselves for a possible future pandemic?
8. Would you say the needs of your older adults in the community have changed following the pandemic? (in what ways? What can be done to improve their situations)
9. Do you have any questions to ask?

Thank you for your time and attention
